# Linking digital teaching management platform quality to perceived net benefits: a hybrid SEM–LPA study in university sports club-based teaching

**DOI:** 10.3389/fpsyg.2026.1903344

**Published:** 2026-08-05

**Authors:** Juntong Liu, Zhihui Zhang

**Affiliations:** Department of Physical Education, Chengdu University of Information Technology, Chengdu, China

**Keywords:** digital teaching management platform, information systems success, latent profile analysis, perceived net benefits, sports club-based teaching, technology acceptance model, university physical education

## Abstract

**Introduction:**

Digital teaching management platforms in university sports club-based teaching coordinate course selection, club allocation, venue use, attendance, assessment, and administrative communication. This study integrated the information systems success model and the technology acceptance model to examine system quality (SQ), perceived ease of use (PEOU), perceived usefulness (PU), user satisfaction (SAT), and perceived teaching management net benefits (NB).

**Methods:**

Data from 1,887 adult platform users were collected in a cross-sectional survey. Structural equation modeling (SEM) estimated sample-wide associations, and latent profile analysis (LPA) identified SQ-PEOU experience configurations. BCH procedures compared PU, SAT, and NB across the profiles.

**Results:**

SEM showed that SQ was positively associated with NB and that PEOU, PU, and SAT formed a significant sequential indirect association. LPA identified six configurations: low-SQ/low-PEOU, moderate-SQ/high-PEOU, low-SQ/high-PEOU, moderate-SQ/low-PEOU, high-SQ/low-PEOU, and high-SQ/high-PEOU. BCH comparisons showed significant differences in PU, SAT, and NB, with the high-SQ/high-PEOU configuration reporting the most favorable evaluations. Profile membership was not significantly associated with role in platform use, years of platform use, frequency of platform use, or main platform function used.

**Discussion:**

Average statistical associations coexisted with heterogeneous SQ-PEOU experience configurations. Because the data were cross-sectional and self-reported, the findings should be interpreted as associations rather than causal processes.

## 1 Introduction

Digital transformation has become a defining agenda in contemporary education. Around the world, universities are increasingly expected to use digital technologies not only to support teaching and learning, but also to improve educational governance, resource coordination, service delivery, and evidence-informed decision making. International organizations have emphasized that educational technology should not be adopted merely because it is available; rather, its value depends on whether it is appropriate, evidence-based, equitable, scalable, and sustainable (UNESCO, 2023). Similarly, the OECD (2023) argues that effective digital education requires a coherent digital ecosystem that connects infrastructure, data, governance, institutional management, and human capacity. In China, the national education digitalization strategy has further accelerated the construction and use of digital platforms across educational sectors, making digital systems an increasingly important infrastructure for educational management and teaching reform (Ministry of Education of the People's Republic of China, 2022).

University physical education provides a particularly demanding setting for digital management. The club-based teaching model gives students greater choice in sport events, learning schedules, participation pathways, and skill-development trajectories, supporting interest, autonomy, and lifelong exercise habits (Gao and Wu, 2004). These benefits also increase coordination requirements involving sport-event choice, club capacity, teacher or coach assignment, venues and time slots, attendance, performance assessment, feedback, and cross-departmental decisions. Unlike a conventional learning management system centered mainly on content delivery, a sports club-based teaching platform must connect movement-based instruction with scarce physical resources and multi-actor administrative workflows. Recent studies have documented the expanding role of digital and intelligent technologies in physical education and their value for teaching, assessment, feedback, and data-informed practice (Martín-Rodríguez and Madrigal-Cerezo, 2025; Zhong et al., 2025), while system-design research has begun to address physical education teaching information management (Rao, 2024; Zhang, 2024).

Evidence nevertheless points to an implementation tension. Digital technologies can support physical education teaching and assessment, yet teachers continue to report constraints involving access to resources, training, institutional support, time, and data privacy (Saiz-González et al., 2025). Taken together, the literature presents an uneven pattern in which reported opportunities for technology-enhanced teaching and assessment coexist with persistent barriers in day-to-day implementation. A platform may therefore be functionally extensive but difficult to operate, easy to navigate but limited in management capacity, or frequently used without being perceived as beneficial. Platform deployment or use alone cannot establish information systems success; evaluation must also consider technical quality, user experience, work processes, and perceived organizational value (Petter et al., 2013). The updated DeLone and McLean information systems success model provides a basis for examining system quality, user satisfaction, and net benefits (DeLone and McLean, 2003), and Wang et al. (2007) demonstrated that these dimensions can be assessed in technology-supported educational and organizational settings.

The relevant literature leaves a clear contextual gap. Much of the digital physical education literature examines instructional technologies, student engagement, movement feedback, or learning outcomes, whereas research on physical education information systems has often emphasized technical design and implementation. Less is known about how adult users evaluate the quality of platforms that organize university sports club-based teaching, how their effort-related and task-related beliefs are associated with satisfaction, and how these evaluations relate to perceived management benefits. This omission matters because net benefits in this setting include the coordination of club capacity, venues, schedules, attendance records, assessment processes, and rapid responses to teaching-management changes, rather than content delivery alone.

A related theoretical gap concerns the connection between platform characteristics and users' evaluations. The information systems success model explains quality, satisfaction, and net benefits, while the technology acceptance model explains perceived ease of use and perceived usefulness as effort-related and performance-related beliefs (Davis, 1989). Integrating the two perspectives makes it possible to examine whether functional system quality is associated with management value both directly and through users' evaluations of operational effort, task usefulness, and satisfaction (Ong et al., 2004; Wixom and Todd, 2005). Applying this integration to sports club-based teaching is theoretically informative because the platform is embedded in a resource-constrained, multi-role management environment rather than a general e-learning setting.

A variable-centered model alone cannot capture the full range of platform experience. SEM estimates whether system quality, ease of use, usefulness, satisfaction, and net benefits are associated on average across the sample, but it does not reveal whether users experience different combinations of functional quality and operational effort. High system quality may coexist with low ease of use, and high ease of use may coexist with limited system quality. LPA complements SEM by identifying unobserved configurations across continuous indicators and by testing whether those configurations differ on theoretically relevant evaluations (Nylund et al., 2007; Spurk et al., 2020). This person-centered evidence can therefore show what the average structural associations conceal.

The present study addresses these gaps by combining an integrated IS success–TAM model with a person-centered analysis of 1,887 users from university sports club-based teaching settings. It makes three contributions. It locates information systems success within a sport-specific management context characterized by club choice, venue constraints, attendance and assessment requirements, and coordination among teachers, coaches, administrators, and technical staff. It clarifies how platform quality, operational effort, task usefulness, satisfaction, and perceived management value are statistically connected without treating cross-sectional associations as causal mechanisms. It also combines SEM and LPA to distinguish sample-wide relationships from system-quality/ease-of-use configurations, providing a stronger basis for differentiated platform evaluation and improvement.

## 2 Literature review and hypothesis development

### 2.1 Theoretical foundations: integrating information systems success and technology acceptance

The updated DeLone and McLean information systems success model conceptualizes system quality, information quality, service quality, use, user satisfaction, and net benefits as interconnected dimensions of system success (DeLone and McLean, 2003). The present study focuses on system quality, satisfaction, and net benefits because they correspond directly to the technical foundation, overall evaluation, and perceived management value of a digital teaching management platform. In sports club-based teaching, system quality concerns whether the platform can sustain course and club selection, allocation, venue coordination, attendance, assessment, communication, and administrative continuity. Wang et al. (2007) showed that these dimensions can be measured meaningfully in technology-supported educational and organizational contexts.

The technology acceptance model adds users' task-related beliefs. Perceived ease of use reflects the effort associated with learning and operating a system, while perceived usefulness reflects the extent to which the system is regarded as supportive of work performance (Davis, 1989). In the present context, these beliefs concern whether teachers, coaches, and administrators can complete recurring management tasks without excessive operational burden and whether the platform helps them perform those tasks effectively. Ong et al. (2004) supported the applicability of these constructs in digitally mediated learning and training settings.

The two perspectives are complementary. System quality represents the functional resources provided by the platform; perceived ease of use represents the effort cost of accessing those resources; perceived usefulness and satisfaction capture cognitive and evaluative responses; and net benefits represent perceived management value. Wixom and Todd (2005) argued that system characteristics, user satisfaction, and technology-acceptance beliefs can be integrated within one explanatory framework. The resulting model is used here to examine theoretically ordered statistical associations, while the cross-sectional design precludes claims about causal or temporal processes.

### 2.2 System quality and teaching management net benefits

System quality may be associated with teaching management net benefits independently of users' intermediate evaluations. Within the information systems success model, system quality captures technical and functional performance, whereas net benefits represent the value perceived from system implementation and use (DeLone and McLean, 2003; Urbach and Müller, 2012). In university sports club-based teaching, those benefits include more efficient coordination of club capacity, venues, schedules, attendance, assessment data, feedback, and administrative responses. Stable access, responsive interfaces, integrated functions, and timely management information may therefore be closely associated with users' assessments of management value. Evidence from e-learning systems and educational management likewise positions system quality as a meaningful basis for evaluating individual and organizational benefits (Pacheco et al., 2025; Wang et al., 2007; Wixom and Todd, 2005). The proposed relationship is treated as a cross-sectional association.

H1. Digital teaching management system quality (SQ) is positively associated with teaching management net benefits (NB).

### 2.3 System quality, perceived ease of use, and perceived usefulness

Digital teaching management system quality (SQ) refers to users' evaluations of platform availability, reliability, interface responsiveness, functional integration, interactivity, information presentation, useful features, and access speed (Secaira et al., 2026). These attributes are directly relevant to course selection, club assignment, attendance recording, venue coordination, performance management, and related administrative tasks in university sports club-based teaching (Zhang, 2024).

System quality is expected to be positively associated with perceived ease of use (PEOU). Stable, accessible, responsive, and integrated functions can reduce the effort required to locate information and complete recurring tasks, whereas fragmented or unreliable functions can make even a feature-rich platform burdensome. This reasoning connects Davis's (1989) effort-related conception of ease of use with the argument that system characteristics shape beliefs about system use (Wang et al., 2007; Wixom and Todd, 2005). Ong et al. (2004) likewise identified ease of use as a central belief in technology acceptance.

H2. Digital teaching management system quality (SQ) is positively associated with perceived ease of use (PEOU).

Perceived ease of use is also expected to be positively associated with perceived usefulness (PU). When learning and operating a platform requires less effort, users can devote more attention to the management task and may more readily recognize the platform's performance value. This association is central to TAM and has been supported in research on the antecedents of usefulness and ease of use (Davis, 1989; Karahanna and Straub, 1999; Venkatesh and Davis, 1996). In sports club-based teaching management, an easier platform can make attendance, allocation, venue, and assessment functions more accessible in daily work (Ong et al., 2004).

H3. Perceived ease of use (PEOU) is positively associated with perceived usefulness (PU).

### 2.4 Perceived usefulness, user satisfaction, and teaching management net benefits

Perceived usefulness (PU) refers to the extent to which users regard the platform as supportive of teaching-management performance. In sports club-based teaching, usefulness is reflected in more efficient information handling, less repetitive administrative work, stronger coordination, and better support for decisions concerning clubs, venues, attendance, and assessment. TAM positions usefulness as a central performance-related belief in users' evaluations of information systems (Davis, 1989; Venkatesh and Davis, 2000), and Ong et al. (2004) confirmed its relevance in an e-learning setting.

User satisfaction (SAT) captures users' overall evaluative response to platform experience. Post-adoption information systems research links satisfaction closely to whether a system meets expectations and remains useful for work (Bhattacherjee, 2001; Vaezi, 2013). Evidence from Chinese teachers' online learning also identifies satisfaction as an important post-adoption evaluation (Meng and Li, 2024). Users who regard the platform as useful for concrete management tasks are therefore expected to report more favorable satisfaction.

H4. Perceived usefulness (PU) is positively associated with user satisfaction (SAT).

Teaching management net benefits (NB) refer to perceived improvements associated with the platform, including work performance, problem solving, responsiveness to management changes, service quality, cost and time savings, shorter processes, and attainment of teaching-management goals. The updated information systems success model treats net benefits as a broad construct spanning individual and organizational value (DeLone and McLean, 2003; Petter et al., 2008; Urbach and Müller, 2012), and Wang et al. (2007) operationalized this construct in an e-learning systems success scale.

Perceived usefulness is expected to be positively associated with net benefits because both concern task performance at different evaluative levels. Usefulness captures whether the platform helps users perform their work, while net benefits capture the broader value they attribute to platform-supported management (DeLone and McLean, 2004; Petter et al., 2008; Wixom and Todd, 2005). This relationship is interpreted as an association rather than a causal pathway.

H5. Perceived usefulness (PU) is positively associated with teaching management net benefits (NB).

User satisfaction is likewise expected to be positively associated with net benefits. The information systems success model treats satisfaction and benefits as distinct but related evaluations of system success (DeLone and McLean, 2003), and Wang et al. (2007) included both dimensions in their e-learning systems framework. Users who evaluate their platform experience favorably may also perceive stronger value for teaching-management work.

H6. User satisfaction (SAT) is positively associated with teaching management net benefits (NB).

### 2.5 Chain indirect relationships among SQ, PEOU, PU, SAT, and NB

The proposed model links platform characteristics, user beliefs, overall evaluation, and perceived management value. SQ represents the technical and functional foundation of the platform; PEOU and PU represent effort-related and performance-related beliefs; SAT represents an overall evaluative response; and NB represents perceived management value. This ordering is consistent with Wixom and Todd's (2005) integration of system characteristics, beliefs, and attitudes and with the connections among quality, satisfaction, and benefits in the updated information systems success model (DeLone and McLean, 2003).

The sequence is tested as a theoretically grounded indirect association. Higher SQ is expected to be associated with greater PEOU, greater PEOU with stronger PU, stronger PU with higher SAT, and higher SAT with greater NB. Because the observations were collected at one time point, support for the sequence would indicate a statistically compatible pattern rather than a demonstrated causal or longitudinal process.

H7. Digital teaching management system quality (SQ) is indirectly associated with teaching management net benefits (NB) through the sequential linkage of perceived ease of use (PEOU), perceived usefulness (PU), and user satisfaction (SAT).

### 2.6 A latent profile perspective on digital teaching management platform experience

The SEM component evaluates average associations and assumes that one population-level structural pattern adequately summarizes the sample. This is useful for estimating how SQ, PEOU, PU, SAT, and NB are related on average, but it cannot show whether users occupy qualitatively different positions across the two foundational experience dimensions. LPA provides a complementary person-centered framework by identifying unobserved subgroups from continuous response patterns and comparing the resulting profiles on external variables (Nylund et al., 2007; Spurk et al., 2020). Its contribution is therefore configurational: it reveals combinations that are not visible in separate means or regression paths.

SQ and PEOU were selected as profile indicators for a theory-driven reason. SQ represents the functional resources supplied by the platform, whereas PEOU reflects the ease with which users can access those resources with limited effort. Their combination can distinguish favorable alignment (high SQ and high PEOU), functional strength accompanied by operational burden (high SQ and low PEOU), ease of operation accompanied by limited functionality (low SQ and high PEOU), and joint disadvantage (low SQ and low PEOU). These patterns are expected to differ in their distal evaluations, even when SQ and PEOU are positively associated in the full sample. Because only two indicators are used, the resulting profiles are interpreted as SQ–PEOU experience configurations rather than comprehensive or stable user typologies.

PU, SAT, and NB were not included as profile indicators because the integrated framework treats them as distal evaluative variables. Keeping them outside the profile-formation process avoids defining profiles partly by the outcomes used to assess their external differentiation. LPA remains exploratory with respect to the number and shape of configurations, so no directional profile hypotheses were specified.

RQ1. What SQ–PEOU experience configurations can be identified from users' perceptions of digital teaching management system quality (SQ) and perceived ease of use (PEOU)?

RQ2. Do the identified SQ–PEOU experience configurations differ in perceived usefulness (PU), user satisfaction (SAT), and teaching management net benefits (NB)?

**Figure 1:**
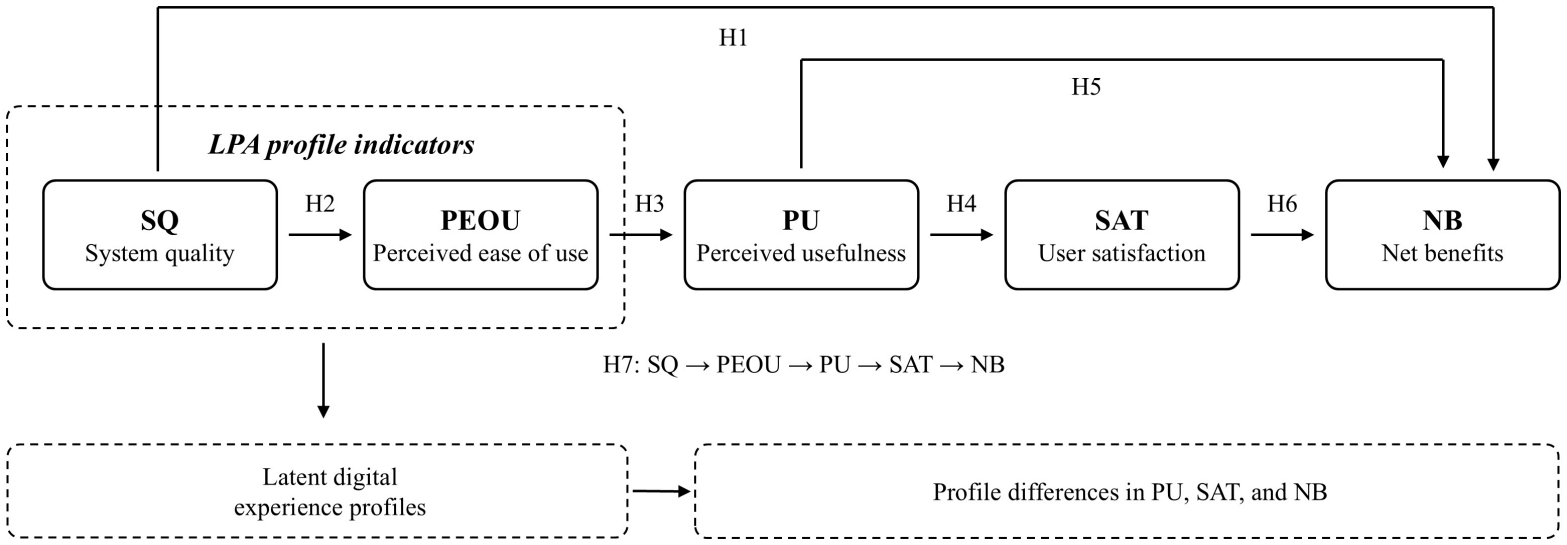
Integrated variable-centered and person-centered analytical model. SQ, digital teaching management system quality; PEOU, perceived ease of use; PU, perceived usefulness; SAT, user satisfaction; NB, perceived teaching management net benefits. The upper component presents the hypothesized SEM associations. The lower component presents the LPA, in which SQ and PEOU form experience configurations and PU, SAT, and NB are examined as distal evaluations.

The resulting design combines two complementary levels of evidence. The SEM component estimates average associations among SQ, PEOU, PU, SAT, and NB. The LPA component identifies SQ–PEOU configurations and uses PU, SAT, and NB as distal evaluations. Figure 1 presents the integrated analytical logic.

## 3 Method

### 3.1 Participants and procedure

This study used a cross-sectional survey design. Participants were recruited from 15 universities in Sichuan Province, China, including Sichuan University, University of Electronic Science and Technology of China, Southwestern University of Finance and Economics, Southwest Jiaotong University, Southwest Petroleum University, Chengdu Technological University, and other universities that had implemented digital teaching management platforms in university sports club-based teaching management. Eligible participants were adult users of the digital teaching management platform, including physical education teachers, sports club instructors or coaches, teaching administrators in physical education departments, academic affairs administrators, platform administrators, and other staff members directly involved in sports club-based teaching management. Students were not included because the present study focused on teaching management net benefits rather than student learning outcomes or student service experience.

The study protocol was reviewed and approved by the Ethics Committee of the Department of Physical Education, Chengdu University of Information Technology, China (Approval No. CUIT2026TY007). Data were collected between January 15 and February 25, 2026. The online questionnaire was distributed through Wenjuanxing, a widely used online survey platform in China. Before entering the formal questionnaire, participants were presented with an informed consent page that explained the study purpose, eligibility criteria, voluntary nature of participation, anonymity of responses, approximate completion time, and the right to withdraw before submission. Only participants who clicked the “I agree” option on the informed consent page were allowed to proceed to the questionnaire. All participants provided written informed consent electronically. No directly identifying personal information, such as names, student/staff IDs, or phone numbers, was collected. Device/IP-related technical information automatically recorded by the survey platform was used only for data-quality screening, was not linked to respondents' identities, and was removed or de-identified before analysis. The data were used only for academic research and were stored in password-protected files accessible only to the research team.

A priori sample size adequacy was evaluated with reference to the requirements of structural equation modeling and latent profile analysis. For SEM, the study involved five latent constructs and 28 observed measurement items. Although minimum sample size requirements in SEM vary depending on model complexity, factor loadings, missing data, and estimation method, simulation evidence suggests that required sample sizes can range widely and that larger samples improve parameter stability and solution propriety (Wolf et al., 2013). Methodological recommendations also suggest that sample size decisions in latent profile analysis should consider model complexity, class separation, classification quality, and the interpretability and size of the smallest class (Nylund et al., 2007; Spurk et al., 2020). The final valid sample of 1,887 participants was therefore considered sufficient for confirmatory factor analysis, structural equation modeling, latent profile analysis, and subsequent profile comparisons.

A total of 2,136 questionnaires were returned. Because all questionnaire items were set as mandatory in Wenjuanxing, there were no missing values in the submitted responses. Data screening was conducted before formal analysis. Responses were excluded if they met any of the following criteria: (a) the respondent did not report direct experience using the digital teaching management platform or did not meet the eligibility criteria; (b) the completion time was less than 180 s, which was considered too short for careful reading and completion of the 28 scale items and demographic questions; (c) the respondent failed the embedded attention-check item, “To confirm that you are reading the items carefully, please select ‘Agree' for this statement”; (d) the response pattern showed insufficient effort, such as selecting the same response option across nearly all scale items or producing an extremely long string of identical responses; or (e) the record appeared to be a duplicate or suspicious submission based on repeated device/IP information and highly similar response timestamps. Specifically, 38 questionnaires were removed because the respondents did not meet the eligibility criteria, 108 because the completion time was below 180 s, 47 because the attention-check item was answered incorrectly, 35 because of insufficient-effort response patterns, and 21 because of duplicate or suspicious submissions. After these exclusions, 1,887 valid questionnaires remained, yielding a valid questionnaire retention rate of 88.34%. The demographic characteristics of the final sample are presented in Table 1.

**Table 1:** Demographic characteristics of the sample (n = 1,887).

Variable	Category	*n*	%
Gender	Male	1,184	62.75
	Female	696	36.88
	Other/Prefer not to say	7	0.37
Age	≤ 30 years	286	15.16
	31–40 years	702	37.20
	41–50 years	621	32.91
	≥ 51 years	278	14.73
Education level	Bachelor's degree or below	354	18.76
	Master's degree	1,186	62.85
	Doctoral degree	347	18.39
Professional title	Teaching assistant	327	17.33
	Lecturer	706	37.41
	Associate professor	589	31.21
	Professor	153	8.11
	Administrative staff/Other professional title	112	5.94
Role in platform use	Physical education teacher	781	41.39
	Teaching administrator	354	18.76
	Club instructor/coach	496	26.29
	Platform administrator	184	9.75
	Other	72	3.82
Years of teaching experience	Less than 5 years	389	20.61
	5–10 years	497	26.34
	11–20 years	641	33.97
	More than 20 years	360	19.08
Years of platform use	Less than 1 year	418	22.15
	1–2 years	699	37.04
	3–4 years	527	27.93
	5 years or more	243	12.88
Frequency of platform use	Rarely	127	6.73
	Several times per month	421	22.31
	Several times per week	823	43.61
	Almost every day	516	27.34
Main platform function used	Course selection management	386	20.46
	Attendance management	462	24.48
	Performance assessment	310	16.43
	Venue/club coordination	344	18.23
	Comprehensive teaching management	385	20.40

### 3.2 Platform context and workflow

Respondents evaluated the digital teaching management platform that they routinely used at their own university. The participating institutions were not confirmed to use a single vendor-specific platform. Vendor identifiers and local-configuration information were not collected systematically; therefore, the number of distinct platform products represented in the sample cannot be determined. The study consequently evaluates a common functional category of sports club-based teaching management platforms rather than a single standardized system. The survey was designed to assess users' evaluations of this common functional category rather than to compare vendor-specific products or interface designs. The contextual description therefore focuses on the management functions, workflow features, and general interface-related quality dimensions covered by the survey.

The functional scope covered by the survey included course and club publication, student selection and capacity management, club allocation, teacher or coach assignment, venue and time-slot coordination, attendance records, performance assessment, feedback, and administrative communication. Because vendor-specific interface information was not collected, interface-related quality was evaluated at a general level through system availability, responsiveness, functional integration, interactivity, information presentation, and information-access speed.

The management workflow represented in the survey encompassed student course or club selection, checks of capacity and scheduling constraints, confirmation of club allocation, recording of attendance and performance, aggregation of assessment and feedback information, and use of the resulting records for teaching coordination. Physical education teachers and club instructors or coaches mainly interacted with attendance, assessment, and feedback functions; teaching administrators handled selection, allocation, schedules, and venue coordination; and platform administrators supported technical operation, user access, and system maintenance. The distribution of the six SQ–PEOU configurations across these role categories was examined in the supplementary subgroup analyses.

### 3.3 Measurements

All constructs were measured using established scales with clear factor structures and prior evidence of reliability and validity in information systems, e-learning, and Chinese educational technology contexts. The questionnaire included five reflective latent constructs: digital teaching management system quality (SQ), perceived ease of use (PEOU), perceived usefulness (PU), user satisfaction (SAT), and teaching management net benefits (NB). During adaptation, references to platform use and work tasks were aligned with sports club-based teaching management, including course selection, club assignment, attendance, venue coordination, performance assessment, feedback, and administrative reporting. Respondents rated each item on a five-point Likert scale ranging from 1 = strongly disagree to 5 = strongly agree. Higher scores indicated higher levels of the corresponding construct. All items were positively worded and scored.

Digital teaching management system quality (SQ) refers to users' perceptions of the technical and functional quality of the digital teaching management platform, including system availability, operational reliability, interface responsiveness, functional integration, interactivity, personalized information presentation, attractive features, and high-speed information access. In this study, SQ was conceptualized as users' evaluation of the platform's technical and functional characteristics rather than their effort-related beliefs about using the platform. SQ was measured using seven items adapted from the system quality dimension of the e-learning systems success scale developed by Wang et al. (2007). This scale was developed based on the updated DeLone and McLean information systems success model and was validated in an organizational e-learning context (DeLone and McLean, 2003; Wang et al., 2007). A representative adapted item is: “The digital teaching management platform provides fast access to the teaching management information I need.”

Perceived ease of use (PEOU) refers to the degree to which users believe that using the digital teaching management platform requires little effort. PEOU was measured using four items adapted from Ong et al. (2004), who modified validated technology acceptance model measures for the asynchronous e-learning context. The theoretical source of this construct is Davis's (1989) technology acceptance model, in which perceived ease of use is defined as the degree to which a person believes that using a system would be free of effort. A representative adapted item is: “I find the digital teaching management platform easy to use.”

Perceived usefulness (PU) refers to the extent to which users believe that using the digital teaching management platform enhances their teaching management performance. PU was measured using four items adapted from Ong et al. (2004), whose e-learning acceptance scale was based on Davis's (1989) technology acceptance model. This construct captures users' perceptions that the platform improves job performance, enhances work effectiveness, increases productivity, and is useful for work-related tasks. A representative adapted item is: “Using the digital teaching management platform improves my effectiveness in sports club-based teaching management.”

User satisfaction (SAT) refers to users' overall affective and evaluative response to their experience with the digital teaching management platform. SAT was measured using three items adapted from the user satisfaction dimension of Wang et al.'s (2007) e-learning systems success scale, with item wording also informed by the information systems continuance literature and Chinese teachers' online learning research (Bhattacherjee, 2001; Meng and Li, 2024). A representative adapted item is: “Overall, I am satisfied with the digital teaching management platform.”

Teaching management net benefits (NB) refer to the perceived benefits associated with the digital teaching management platform for sports club-based teaching management, including improved work performance, better problem solving, faster responses to management changes, improved service quality, cost saving, shortened management processes, and achievement of teaching management goals. NB was measured using ten items adapted from the net benefits dimension of Wang et al.'s (2007) e-learning systems success scale. The net benefits construct is consistent with the updated information systems success model, which integrates individual and organizational impacts into a broader benefits construct (DeLone and McLean, 2003). A representative adapted item is: “The digital teaching management platform helps improve my teaching management performance.”

### 3.4 Data analysis

Data analysis was conducted using SPSS 29.0, AMOS 26.0, and Mplus 8.3. SPSS was used for data coding, screening, descriptive statistics, internal consistency analysis, bivariate correlations, and supplementary chi-square tests. AMOS was used for confirmatory factor analysis (CFA), structural equation modeling (SEM), and bootstrap estimation of indirect associations. Mplus was used for latent profile analysis (LPA) and BCH comparisons of distal variables.

Common-method variance was evaluated because the substantive variables were obtained from a single self-report questionnaire. Harman's single-factor exploratory factor analysis was used as an initial diagnostic. A one-factor CFA, in which all measurement items loaded on one latent factor, was also estimated. These procedures were treated as diagnostic evidence rather than as proof that common-method variance was absent (Podsakoff et al., 2003). CFA and SEM fit were evaluated using χ^2^/*df* , CFI, TLI, SRMR, and RMSEA, with multiple indices considered jointly (Hu and Bentler, 1999; Kline, 2016).

The measurement model was assessed before the structural model. Internal consistency was evaluated using Cronbach's α and composite reliability (CR), with values of 0.70 or higher considered acceptable (Bagozzi and Yi, 1988; Nunnally and Bernstein, 1994). Convergent validity was assessed from standardized factor loadings and average variance extracted (AVE), using 0.50 as the minimum criterion for loadings and AVE (Fornell and Larcker, 1981; Hair et al., 2019). Discriminant validity was examined with the Fornell–Larcker criterion, and Pearson correlations among composite mean scores were reported as preliminary bivariate associations.

SEM was then performed to evaluate H1–H7. Model adequacy was assessed using χ^2^/*df* , CFI, TLI, SRMR, and RMSEA. Standardized path coefficients and two-tailed significance levels were reported for the hypothesized direct associations. AMOS standard errors and critical ratios are calculated for unstandardized estimates and were therefore not combined with standardized coefficients in Table 2. The model reported in the Results did not include demographic or platform-use control variables.

**Table 2:** Structural path estimates of the hypothesized SEM model.

Path	Standardized β	*p*	Decision
SQ → NB	0.313	< 0.001	H1 supported
SQ → PEOU	0.361	< 0.001	H2 supported
PEOU → PU	0.491	< 0.001	H3 supported
PU → SAT	0.444	< 0.001	H4 supported
PU → NB	0.211	< 0.001	H5 supported
SAT → NB	0.231	< 0.001	H6 supported

β, standardized path coefficient; SQ, digital teaching management system quality; PEOU, perceived ease of use; PU, perceived usefulness; SAT, user satisfaction; NB, perceived teaching management net benefits. All *p* values are two-tailed.

Bootstrap analysis with 2,000 resamples was used to estimate direct, specific indirect, sequential indirect, total indirect, and total associations from SQ to NB. Bias-corrected 95% confidence intervals that excluded zero were taken as evidence of a statistically supported indirect association (Hayes and Scharkow, 2013; Preacher and Hayes, 2008). The cross-sectional estimates were not interpreted as causal mediation.

LPA was conducted in Mplus 8.3 using the composite mean scores of SQ and PEOU. One- through seven-profile solutions were estimated with robust maximum likelihood (MLR). In the conventional diagonal LPA specification used here, indicator means were freely estimated across profiles, indicator variances were constrained equal across profiles, and within-profile indicator covariances were fixed at zero. Multiple random starting values were used, and solutions were retained only when the best log-likelihood was replicated. Model selection considered log-likelihood, AIC, BIC, sample-size adjusted BIC (aBIC), entropy, the Lo–Mendell–Rubin adjusted likelihood ratio test (LMR), the bootstrap likelihood ratio test (BLRT), profile proportions, parsimony, and substantive interpretability (Nylund et al., 2007; Spurk et al., 2020).

PU, SAT, and NB were specified as distal variables rather than profile indicators. The BCH method was used to compare their adjusted means across profiles while accounting for classification uncertainty (Asparouhov and Muthén, 2021; Dziak et al., 2016). Wald χ^2^ tests evaluated overall differences, followed by BCH-adjusted pairwise comparisons. This separation preserved the distinction between profile formation from SQ and PEOU and external evaluation through PU, SAT, and NB.

Exploratory Pearson chi-square tests were also conducted using most likely profile membership in the selected six-profile solution to examine its association with role in platform use, years of platform use, frequency of platform use, and main platform function used. Cramér's V was reported as the effect-size index, and expected cell counts were inspected. These analyses were supplementary and descriptive rather than predictors of latent profile membership. All statistical tests used a two-tailed significance level of *p* < 0.05.

## 4 Results

### 4.1 Common-method variance diagnostics

Harman's single-factor analysis of the 28 measurement items yielded five factors with eigenvalues greater than 1. Together, these factors explained 66.624% of the total variance, and the first factor accounted for 34.376%.

A one-factor CFA, in which all 28 items were specified to load on a single latent factor, showed poor fit to the data, χ^2^(350) = 13,530.382, *p* < 0.001, χ^2^/*df* = 38.658, CFI = 0.551, TLI = 0.515, SRMR = 0.132, and RMSEA = 0.141, 90% CI [0.139, 0.143]. The Harman test and one-factor CFA did not support an explanation in which a single common factor accounted for the covariance among the items. These diagnostics cannot completely rule out common-method variance, because all substantive variables were collected from the same respondents at one time point; the findings are therefore interpreted as associations based on self-reported evaluations.

### 4.2 Measurement model, descriptive statistics, reliability, and validity

The hypothesized five-factor measurement model showed good fit to the data, χ^2^(340) = 374.728, *p* = 0.094, χ^2^/*df* = 1.102, CFI = 0.999, TLI = 0.999, SRMR = 0.013, and RMSEA = 0.007, 90% CI [0.000, 0.012]. The results supported the empirical distinction among SQ, PEOU, PU, SAT, and NB.

Table 3 presents the descriptive statistics, internal consistency reliability, and convergent validity results. Construct means ranged from 3.231 to 3.591, and standard deviations ranged from 0.907 to 1.042. Cronbach's α values ranged from 0.802 to 0.930, and CR values ranged from 0.802 to 0.931. Standardized factor loadings ranged from 0.707 to 0.882, and AVE values ranged from 0.575 to 0.657. These results supported the internal consistency reliability and convergent validity of the five constructs.

**Table 3:** Descriptive statistics, internal consistency reliability, and convergent validity of the study constructs.

Variable	M	SD	Cronbach's α	Std. factor loading	CR	AVE
SQ	3.582	0.943	0.910	0.741–0.849	0.911	0.595
PEOU	3.388	1.042	0.883	0.752–0.882	0.884	0.657
PU	3.231	1.010	0.854	0.707–0.812	0.854	0.595
SAT	3.358	1.010	0.802	0.745–0.774	0.802	0.575
NB	3.591	0.907	0.930	0.722–0.795	0.931	0.575

M, mean; SD, standard deviation; CR, composite reliability; AVE, average variance extracted; Std. factor loading, standardized factor-loading range obtained from the five-factor confirmatory factor analysis. SQ, digital teaching management system quality; PEOU, perceived ease of use; PU, perceived usefulness; SAT, user satisfaction; NB, perceived teaching management net benefits.

All composite-score correlations were positive and statistically significant, ranging from *r* = 0.274 to *r* = 0.412, all *p* < 0.001. The strongest associations were observed between SQ and NB, *r* = 0.412, and between PEOU and PU, *r* = 0.402. The square roots of AVE ranged from 0.758 to 0.810 and exceeded the corresponding composite-score correlations, supporting discriminant validity under the Fornell–Larcker criterion (Table 4).

**Table 4:** Composite-score correlations and discriminant validity of the study variables.

Variable	SQ	PEOU	PU	SAT	NB
SQ	0.772				
PEOU	0.308^***^	0.810			
PU	0.365^***^	0.402^***^	0.772		
SAT	0.274^***^	0.307^***^	0.355^***^	0.758	
NB	0.412^***^	0.333^***^	0.367^***^	0.353^***^	0.758

^***^p < 0.001.

Diagonal values in bold are the square roots of AVE; off-diagonal values are Pearson correlations based on composite mean scores. SQ, digital teaching management system quality; PEOU, perceived ease of use; PU, perceived usefulness; SAT, user satisfaction; NB, perceived teaching management net benefits.

### 4.3 Structural equation modeling and bootstrap analysis of indirect associations

The structural equation model showed good fit to the data, χ^2^(344) = 620.271, *p* < 0.001, χ^2^/*df* = 1.803, CFI = 0.991, TLI = 0.990, SRMR = 0.064, and RMSEA = 0.021, 90% CI [0.018, 0.023].

All hypothesized direct associations were positive and statistically significant (Table 2). Digital teaching management system quality (SQ) was positively associated with teaching management net benefits (NB; β = 0.313, *p* < 0.001), supporting H1. SQ was also positively associated with perceived ease of use (PEOU; β = 0.361, *p* < 0.001), supporting H2. PEOU was positively associated with perceived usefulness (PU; β = 0.491, *p* < 0.001), supporting H3. PU was positively associated with user satisfaction (SAT; β = 0.444, *p* < 0.001), supporting H4. In addition, PU (β = 0.211, *p* < 0.001) and SAT (β = 0.231, *p* < 0.001) were positively associated with NB, supporting H5 and H6, respectively.

**Figure 2:**
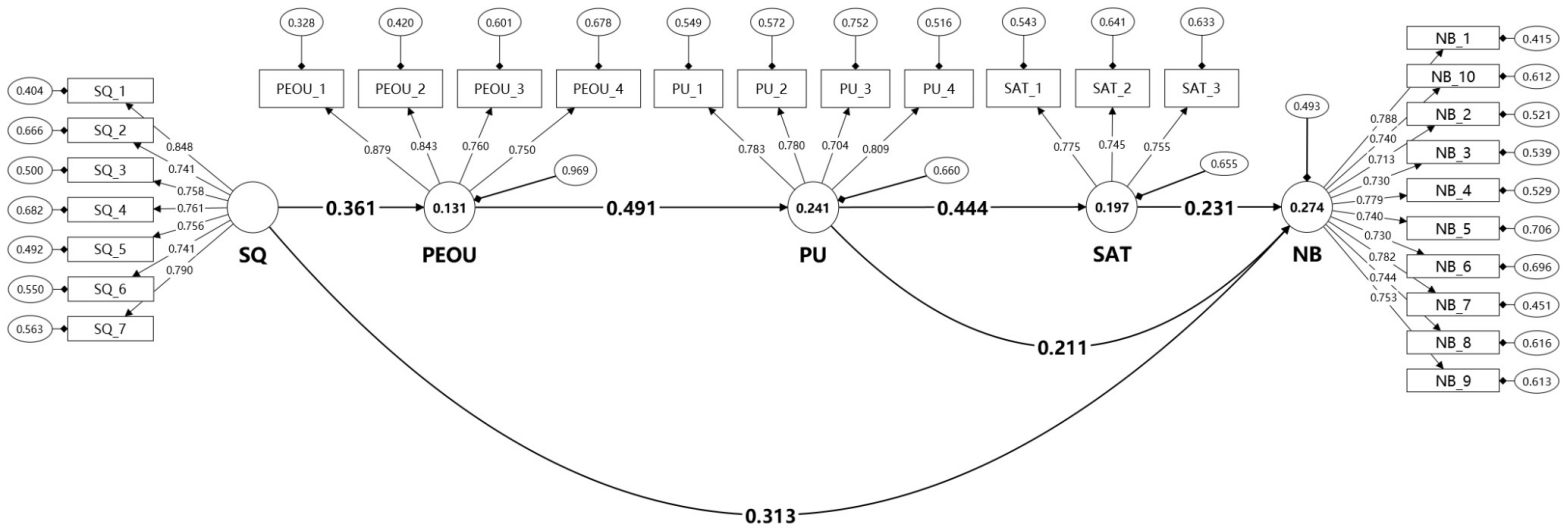
Standardized path coefficients of the structural equation model. Standardized path coefficients are shown on the structural paths, and *R*^2^ values are displayed above the endogenous constructs. All displayed paths were statistically significant at *p* < 0.001. SQ, digital teaching management system quality; PEOU, perceived ease of use; PU, perceived usefulness; SAT, user satisfaction; NB, perceived teaching management net benefits.

The model accounted for 13.1% of the variance in PEOU, 24.1% of the variance in PU, 19.7% of the variance in SAT, and 27.4% of the variance in NB. Figure 2 presents the standardized structural coefficients and *R*^2^ values.

As shown in Table 5, bootstrap analysis with 2,000 resamples showed a significant direct association between SQ and NB, β = 0.313, Boot SE = 0.025, 95% CI [0.261, 0.360]. The specific indirect association through PEOU and PU was significant, β = 0.037, Boot SE = 0.007, 95% CI [0.025, 0.051], as was the sequential indirect association through PEOU, PU, and SAT, β = 0.018, Boot SE = 0.003, 95% CI [0.013, 0.025]. The total indirect association was β = 0.056, Boot SE = 0.007, 95% CI [0.042, 0.070], and the total association was β = 0.369, Boot SE = 0.024, 95% CI [0.318, 0.414]. Because the confidence interval for the sequential indirect association excluded zero, H7 was supported. These estimates represent statistically supported indirect associations and do not establish causal mediation or temporal ordering.

**Table 5:** Bootstrap analysis of direct, indirect, and total associations between SQ and NB.

Association type	Path	β	Boot SE	Boot LLCI	Boot ULCI
Direct association	SQ → NB	0.313	0.025	0.261	0.360
Indirect association	SQ → PEOU → PU → NB	0.037	0.007	0.025	0.051
Indirect association	SQ → PEOU → PU → SAT → NB	0.018	0.003	0.013	0.025
Total indirect association	All indirect paths from SQ to NB	0.056	0.007	0.042	0.070
Total association	SQ → NB	0.369	0.024	0.318	0.414

β, standardized estimate; Boot SE = bootstrap standard error; Boot LLCI, lower limit of the 95% bootstrap confidence interval; Boot ULCI, upper limit of the 95% bootstrap confidence interval. Bootstrap sample size = 2,000. An association was considered statistically significant when its 95% confidence interval did not include zero. SQ, digital teaching management system quality; PEOU, perceived ease of use; PU, perceived usefulness; SAT, user satisfaction; NB, perceived teaching management net benefits.

### 4.4 Latent profile analysis and distal variable differences

LPA used the composite mean scores of SQ and PEOU as profile indicators. The resulting profiles are interpreted as SQ–PEOU experience configurations rather than comprehensive user typologies. One- through seven-profile solutions were compared (Table 6). The six-profile solution had the lowest BIC (9,872.418), acceptable classification quality (entropy = 0.774), significant LMR and BLRT results (both *p* < 0.001), and profile proportions ranging from 12.45% to 31.80%. Although the seven-profile solution produced slightly lower AIC and aBIC values, its BIC increased, the LMR test was not significant (*p* = 0.147), and one profile contained only 3.50% of the sample. The seven-profile solution also offered no clear gain in substantive interpretability. The six-profile solution was therefore retained on the basis of model fit, classification quality, profile size, parsimony, and interpretability.

**Table 6:** Fit indices and classification information for latent profile models with one to seven profiles.

Model	LL	AIC	BIC	aBIC	Entropy	LMR p	BLRT p	Profile proportions (%)
1-profile model	−5,320.801	10,649.603	10,671.774	10,659.066	—	—	—	100.00
2-profile model	−5,073.412	10,160.823	10,199.623	10,177.384	0.782	< 0.001	< 0.001	43.77/56.23
3-profile model	−5,006.013	10,032.027	10,087.454	10,055.684	0.789	< 0.001	< 0.001	44.30/26.71/28.99
4-profile model	−4,922.504	9,871.009	9,943.065	9,901.764	0.773	< 0.001	< 0.001	20.30/22.84/19.61/37.26
5-profile model	−4,906.792	9,845.583	9,934.267	9,883.435	0.757	0.005	< 0.001	15.95/11.61/22.47/12.40/37.57
6-profile model (selected)	−4,864.553	9,767.106	9,872.418	9,812.055	0.774	< 0.001	< 0.001	14.04/14.89/12.93/13.88/ 12.45/31.80
7-profile model	−4,856.465	9,756.930	9,878.871	9,808.977	0.771	0.147	< 0.001	12.08/29.15/3.50/14.31/14.10/ 14.20/12.67

LL, log-likelihood; AIC, Akaike information criterion; BIC, Bayesian information criterion; aBIC, sample-size adjusted BIC; LMR, Lo–Mendell–Rubin adjusted likelihood ratio test; BLRT, bootstrap likelihood ratio test. Lower information criteria indicate better relative fit, whereas higher entropy indicates greater classification precision. LMR and BLRT compare the k-profile solution with the k – 1 solution. Profile proportions are based on most likely profile membership and are reported in profile-number order. The LPA indicators were the composite mean scores of SQ and PEOU.

**Figure 3:**
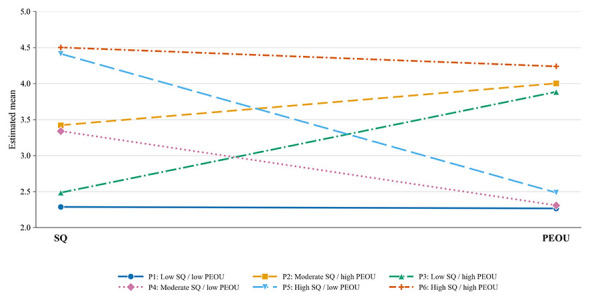
Estimated means of SQ and PEOU across the selected six-profile solution. SQ, digital teaching management system quality; PEOU, perceived ease of use. Values are estimated raw means of the two profile indicators, calculated as composite mean scores on a five-point scale. Profile labels refer to the relative levels of SQ and PEOU within each profile.

The six retained configurations are displayed in Figure 3. Profile 1 showed low SQ and low PEOU; Profile 2, moderate SQ and high PEOU; Profile 3, low SQ but high PEOU; Profile 4, moderate SQ but low PEOU; Profile 5, high SQ but low PEOU; and Profile 6, high SQ and high PEOU. Their estimated indicator means were 2.287/2.266, 3.421/4.002, 2.485/3.884, 3.340/2.308, 4.413/2.485, and 4.501/4.238 for SQ/PEOU, respectively. These results answered RQ1 by identifying six distinct SQ–PEOU experience configurations.

Profile 6 was the largest configuration, comprising 600 participants (31.80%). Profiles 1–5 contained 265 (14.04%), 281 (14.89%), 244 (12.93%), 262 (13.88%), and 235 (12.45%) participants, respectively. Average posterior probabilities ranged from 0.759 to 0.899, indicating acceptable classification precision (Table 7).

**Table 7:** Profile membership and average posterior probabilities for the selected six-profile solution.

Profile	Profile label	*n*	%	Average posterior probability	Interpretation
Profile 1	Low-SQ/low-PEOU profile	265	14.04	0.877	Low SQ and low PEOU
Profile 2	Moderate-SQ/high-PEOU profile	281	14.89	0.770	Moderate SQ and high PEOU
Profile 3	Low-SQ/high-PEOU profile	244	12.93	0.814	Low SQ but high PEOU
Profile 4	Moderate-SQ/low-PEOU profile	262	13.88	0.759	Moderate SQ but low PEOU
Profile 5	High-SQ/low-PEOU profile	235	12.45	0.840	High SQ but low PEOU
Profile 6	High-SQ/high-PEOU profile	600	31.80	0.899	High SQ and high PEOU

SQ, digital teaching management system quality; PEOU, perceived ease of use. Values of n and percentages are based on most likely profile membership. Profile labels summarize the relative levels of SQ and PEOU. Percentages may not sum to 100 because of rounding.

BCH analyses compared PU, SAT, and NB across the six configurations while accounting for classification uncertainty (Table 8). Significant overall differences were found for PU, Wald χ^2^(5) = 488.147, *p* < 0.001; SAT, Wald χ^2^(5) = 249.118, *p* < 0.001; and NB, Wald χ^2^(5) = 539.166, *p* < 0.001. Profile 6 had the highest adjusted mean on all three distal variables, whereas Profile 1 had the lowest. For PU, Profile 2 ranked below Profile 6, Profiles 3 and 5 did not differ from each other, and Profile 4 was lower. For SAT, Profile 2 exceeded Profiles 4 and 5, whereas Profile 3 did not differ significantly from Profiles 2, 4, or 5. For NB, Profiles 2 and 5 formed the next higher group, Profiles 3 and 4 formed a lower-middle group, and Profile 1 remained the lowest. These results answered RQ2 by showing systematic distal-variable differences across the SQ–PEOU experience configurations.

**Table 8:** BCH-adjusted distal variable differences across the six SQ–PEOU experience configurations.

Profile	PU M ±SE	SAT M ±SE	NB M ±SE
Profile 1: Low-SQ/low-PEOU profile	2.451 ± 0.062^e^	2.740 ± 0.067^d^	2.863 ± 0.053^d^
Profile 2: Moderate-SQ/high-PEOU profile	3.381 ± 0.079^b^	3.484 ± 0.076^b^	3.638 ± 0.071^b^
Profile 3: Low-SQ/high-PEOU profile	3.095 ± 0.075^c^	3.255 ± 0.080^bc^	3.352 ± 0.073^c^
Profile 4: Moderate-SQ/low-PEOU profile	2.803 ± 0.077^d^	3.071 ± 0.082^c^	3.242 ± 0.072^c^
Profile 5: High-SQ/low-PEOU profile	3.041 ± 0.075^c^	3.206 ± 0.081^c^	3.678 ± 0.068^b^
Profile 6: High-SQ/high-PEOU profile	3.846 ± 0.040^a^	3.819 ± 0.042^a^	4.128 ± 0.034^a^
Wald χ^2^	488.147	249.118	539.166
df	5	5	5
*p*	< 0.001	< 0.001	< 0.001

PU, perceived usefulness; SAT, user satisfaction; NB, teaching management net benefits; SE, standard error. The BCH method accounts for uncertainty in latent profile classification. Superscript letters represent BCH-adjusted pairwise comparisons within each column. Means that do not share a superscript letter differ significantly at *p* < 0.05; means sharing at least one letter do not differ significantly.

### 4.5 Supplementary subgroup analyses of profile membership

Exploratory Pearson chi-square tests were conducted to examine whether most likely profile membership in the selected six-profile solution was associated with role in platform use, years of platform use, frequency of platform use, or main platform function used. All expected cell counts exceeded five, with a minimum expected count of 8.97 across the four analyses. Detailed subgroup distributions are reported in Supplementary Table 1.

Profile membership was not significantly associated with role in platform use, χ^2^(20) = 12.202, *p* = 0.909, Cramér's *V* = 0.040; years of platform use, χ^2^(15) = 12.915, *p* = 0.609, Cramér's V = 0.048; frequency of platform use, χ^2^(15) = 19.875, *p* = 0.177, Cramér's *V* = 0.059; or main platform function used, χ^2^(20) = 22.154, *p* = 0.332, Cramér's *V* = 0.054. The effect-size estimates were uniformly small.

Profile 6, the high-SQ/high-PEOU profile, was descriptively the largest profile within every subgroup category. However, the overall profile distributions were broadly comparable across categories of role in platform use, years of platform use, frequency of platform use, and main platform function used. The supplementary analyses therefore provided no evidence that the six SQ–PEOU profiles were concentrated within particular observed user-role or platform-use groups.

## 5 Discussion

### 5.1 Overall interpretation of the findings

This study examined platform success in university sports club-based teaching through both average associations and heterogeneous experience configurations. The SEM results showed that perceived net benefits were associated with system quality, operational ease, task usefulness, and satisfaction, while the LPA results showed that users combined system quality and ease of use in distinct ways. Taken together, the findings shift attention from platform deployment to the quality of the management experience created around club allocation, venues, schedules, attendance, assessment, and communication (Aparicio et al., 2016; Hassanzadeh et al., 2012).

The direct association between SQ and NB indicates that users' judgments of technical and functional quality were closely connected with perceived management value. This connection is especially plausible in sports club-based teaching, where delayed access, fragmented modules, unstable attendance functions, or weak information integration can disrupt coordination of people, venues, and time-sensitive activities. The significant indirect associations also show that system quality was linked to net benefits through effort-related, usefulness, and satisfaction evaluations. Platform success in this setting therefore includes both concrete functional performance and the experience of completing management work (Al-Fraihat et al., 2020; Chopra et al., 2019).

The statistical ordering should remain separate from causal interpretation. The data were collected once from the same respondents, so the model does not establish that system quality preceded ease of use, usefulness, satisfaction, or benefits. The supported direct and indirect estimates represent theoretically coherent associations, not evidence of a longitudinal mechanism (Maxwell and Cole, 2007; Maxwell et al., 2011; Rohrer, 2018).

### 5.2 Associations within the integrated IS success–TAM model

The SEM findings support the integration of IS success and TAM in a management-oriented physical education setting. SQ was positively associated with PEOU, and PEOU was positively associated with PU. A stable and integrated platform can reduce the effort required to locate information or complete recurring procedures, while lower operational effort can make its task value more apparent. This extends the familiar TAM beliefs into work that depends on real-time coordination of clubs, venues, attendance, and assessment rather than on content access alone (Davis, 1989; Scherer et al., 2019).

PU was positively associated with both SAT and NB, and SAT was positively associated with NB. Usefulness in this context refers to fit with concrete management requirements: reducing repetitive entry, supporting allocation and coordination, and making records available when decisions are required. Satisfaction captures the overall evaluation of that experience. The pattern is consistent with post-adoption research in which usefulness and satisfaction remain closely related (Bhattacherjee, 2001; Hong et al., 2006; Oghuma et al., 2016) and with Wixom and Todd's (2005) argument that system characteristics and acceptance evaluations should be examined together.

The results also delimit what technical improvement can accomplish on its own. Functional expansion may strengthen perceptions of system quality, but it will not necessarily produce equally favorable usefulness or satisfaction when workflows remain effortful. Conversely, a smooth interface may improve daily experience without supplying the functional depth needed for venue, allocation, attendance, and assessment management. The LPA findings make this distinction visible.

### 5.3 From average associations to experience configurations: Integrating SEM and LPA findings

SEM established a positive sample-wide relationship between SQ and PEOU, but the six-profile solution showed that the two dimensions were not interchangeable. Profile 6, the high-SQ/high-PEOU profile, reported the most favorable PU, SAT, and NB, whereas Profile 1, the low-SQ/low-PEOU profile, reported the least favorable evaluations. These aligned profiles are consistent with the structural results: favorable platform resources and ease of operation tend to occur with more favorable distal evaluations.

The mixed configurations provide the added theoretical value. Profile 5, the high-SQ/low-PEOU profile, still reported relatively high NB, suggesting that strong functionality can support management value even when operation remains burdensome. Its PU and SAT were less favorable than those of Profile 2, the moderate-SQ/high-PEOU profile. From a task–technology fit perspective, functional capacity can support the requirements of venue coordination, attendance, and assessment, while TAM indicates that effortful operation can weaken users' evaluations of usefulness and satisfaction (Davis, 1989; Goodhue and Thompson, 1995). Profile 3, the low-SQ/high-PEOU profile, showed the reverse configuration: ease of use was accompanied by more favorable evaluations than in the low-SQ/low-PEOU profile, but limited SQ constrained the broader management value that users perceived.

The profiles therefore explain why an average positive path does not imply a uniform user experience. SEM indicates the general direction and strength of associations; LPA identifies the combinations through which those associations are instantiated. BCH comparisons then show that the combinations have different external evaluations while accounting for classification uncertainty (Dziak et al., 2016; Spurk et al., 2020). The supplementary chi-square tests add a further point: profile membership was not significantly associated with role in platform use, years of platform use, frequency of platform use, or main platform function used. The profiles cannot be inferred reliably from these observed characteristics alone, which supports direct assessment of users' perceptions of SQ and PEOU rather than assigning needs from demographic or usage labels.

### 5.4 Theoretical implications

The study contributes to information systems success research by specifying the management demands of university sports club-based teaching. Platform quality in this setting concerns the coordination of sport choices, club capacity, venues, schedules, instructors, attendance, assessment, and communication. This differs from a general e-learning context in which content delivery is often central. Recent physical education technology research has expanded rapidly, but much of it remains focused on instruction, engagement, and learning tools (Martín-Rodríguez and Madrigal-Cerezo, 2025; Zhong et al., 2025). The present evidence extends the discussion to staff-oriented digital management and perceived organizational value.

The integrated model clarifies the roles of functional resources and operational effort. SQ was associated with NB directly and through PEOU, PU, and SAT. This pattern indicates that technical quality and user evaluation are connected but analytically distinct. In sports club-based teaching management, users can recognize concrete value from a functionally capable system while still reporting operational burden, a possibility made visible by the high-SQ/low-PEOU configuration. The findings therefore refine IS success–TAM integration by showing how the same constructs operate within complex physical-resource and multi-role workflows (Wixom and Todd, 2005).

The hybrid design contributes a configuration perspective. The profiles are not presented as deep or permanent user types; they are empirically identified combinations of SQ and PEOU. Their value lies in demonstrating that functionally similar or operationally similar experiences can be accompanied by different distal evaluations. Combining SEM, LPA, and BCH thus provides evidence at three levels: average structural associations, heterogeneous foundational configurations, and differences in distal evaluations. This layered evidence is the principal methodological and theoretical contribution of the study.

### 5.5 Practical implications

Universities should evaluate digital teaching management reform through the quality of task support rather than platform deployment alone. Core indicators should include stability, response speed, information accuracy, module integration, and the timely availability of data for club allocation, venues, schedules, attendance, assessment, and communication. Recent physical education system-design research likewise highlights the management value of integrated information functions (Rao, 2024).

Quality and usability require parallel improvement. The high-SQ/low-PEOU configuration shows that adding functions without simplifying workflows can leave users with substantial operational burden. Universities and developers should reduce unnecessary steps, standardize navigation, improve interface consistency, provide task-oriented dashboards, and test routine workflows with the staff who perform them. Human-centered design remains relevant because users' needs, tasks, and contexts should guide interactive-system development (International Organization for Standardization, 2019).

Improvement priorities can be organized around the identified profiles. The low-SQ/low-PEOU profile requires foundational work on reliability, information quality, workflow clarity, and practical support. The low-SQ/high-PEOU profile requires stronger functions and data integration, whereas the moderate-SQ/low-PEOU and high-SQ/low-PEOU profiles require workflow redesign and targeted usability support. The moderate-SQ/high-PEOU profile may benefit most from advanced functions that preserve its operational simplicity. The high-SQ/high-PEOU profile can serve as a source of positive workflow examples and continuous feedback. Because the subgroup analyses were not significant, role in platform use or frequency of platform use should not be used as a proxy for profile membership.

Role-sensitive access can still improve task fit without assuming that all members of a role share the same profile. Teachers and coaches need efficient attendance, assessment, and feedback functions; teaching administrators need selection, allocation, schedule, venue, and exception-management tools; and platform administrators need reliable access management, monitoring, maintenance, and support functions. Evaluation should combine user surveys with system logs, helpdesk records, processing times, coordination errors, and data-completeness indicators. Such evidence would distinguish perceived value from objective management performance and provide a stronger basis for platform revision.

### 5.6 Limitations and future research

The cross-sectional design does not establish temporal order among SQ, PEOU, PU, SAT, and NB. The sequential indirect association is theoretically informative, but it cannot demonstrate that change in one construct produced later change in another. Longitudinal, cross-lagged, or intervention-based designs are needed to examine temporal processes and to avoid treating cross-sectional mediation-like estimates as longitudinal mechanisms (Maxwell et al., 2011).

All substantive variables were self-reported by the same respondents. Procedural safeguards, Harman's test, and the one-factor CFA reduce concern about a dominant single factor, but they do not eliminate common-method variance or subjective response tendencies. Future research should integrate platform logs, administrative records, helpdesk data, processing time, attendance completeness, coordination errors, and other observable management indicators.

The sample was drawn from universities in Sichuan Province and included adult staff users rather than students. The participating institutions were not confirmed to use a single vendor-specific platform. Vendor-specific product, interface, and local-configuration information was not systematically collected, so the number of platform products represented could not be determined and vendor- or interface-level differences could not be examined. The findings therefore concern a common functional category of sports club-based teaching management platforms. Future studies should compare regions, university types, platform vendors, and implementation arrangements and should distinguish staff-oriented management benefits from student-facing learning and service outcomes.

Institution-level clustering and platform-level implementation heterogeneity were not modeled. Respondents from the same university may have shared platform configurations, training arrangements, technical support, and implementation conditions, which may have induced within-institution dependence. Future research should explicitly retain and model institutional identifiers through multilevel or cluster-robust SEM and mixture-modeling approaches.

The LPA was intentionally based on SQ and PEOU as the two profile indicators, with PU, SAT, and NB reserved as distal variables. This design provides a clear test of configuration differences, but the profiles should not be interpreted as comprehensive user typologies. Because the profile model used two continuous indicators, the six-profile solution may partly reflect a statistical partitioning of the joint SQ–PEOU distribution into level combinations rather than qualitatively discrete latent subpopulations. Richer profile models could include information quality, service quality, digital competence, organizational support, training availability, or actual use intensity. The supplementary subgroup tests also relied on most likely profile assignments and should be interpreted as exploratory descriptions rather than formal covariate models.

The six-profile solution is sample-dependent and requires replication. Entropy and posterior probabilities supported acceptable rather than perfect classification, and alternative samples may yield different numbers or shapes of profiles. Replication across institutions, confirmatory mixture analyses, and latent transition models could test whether the configurations remain stable and whether users move toward more favorable configurations after workflow redesign, training, or technical improvement.

## 6 Conclusion

This study showed that perceived system quality, ease of use, usefulness, satisfaction, and teaching management net benefits were positively connected in university sports club-based teaching management. SEM identified sample-wide direct and indirect associations, while LPA revealed six SQ–PEOU experience configurations with distinct usefulness, satisfaction, and net-benefit evaluations. The high-SQ/high-PEOU profile was the most favorable, whereas the low-SQ/low-PEOU profile was the least favorable. Profile membership was broadly distributed across categories of role in platform use and platform-use characteristics. Digital reform in university physical education should therefore assess whether platform functions and workflows jointly support club allocation, venue coordination, attendance, assessment, communication, and administrative decisions. The findings remain cross-sectional and self-reported and should be interpreted as statistical associations rather than causal processes.

## Data Availability

The original contributions presented in the study are included in the article/Supplementary material, further inquiries can be directed to the corresponding author.
