# Supplementary material for: Linking digital teaching management platform quality to perceived net benefits: a hybrid SEM–LPA study in university sports club-based teaching
Source: Front Psychol. 2026 Aug 5;17:1903344. doi: 10.3389/fpsyg.2026.1903344 (PMC13485549; doi:10.3389/fpsyg.2026.1903344)
Supplement: Supplementary file 1 [file Table_1.docx]

**Supplementary Table S1. Distribution of the six SQ–PEOU profiles by user role and platform-use characteristics**

Cells report n (row %), based on most likely profile membership in the selected six-profile solution.

| **Subgroup** | **χ²** | **df** | **p** | **Cramér’s V** | **Minimum expected count** | **Conclusion** |
| --- | --- | --- | --- | --- | --- | --- |
| Role in platform use | 12.202 | 20 | .909 | .040 | 8.97 | No significant association |
| Years of platform use | 12.915 | 15 | .609 | .048 | 30.26 | No significant association |
| Frequency of platform use | 19.875 | 15 | .177 | .059 | 15.82 | No significant association |
| Main platform function used | 22.154 | 20 | .332 | .054 | 38.61 | No significant association |

**Panel A. Profile membership by role in platform use**

| **Category** | **Profile 1** | **Profile 2** | **Profile 3** | **Profile 4** | **Profile 5** | **Profile 6** | **Total** |
| --- | --- | --- | --- | --- | --- | --- | --- |
| Physical education teacher | 95 (12.2%) | 124 (15.9%) | 109 (14.0%) | 106 (13.6%) | 94 (12.0%) | 253 (32.4%) | 781 |
| Teaching administrator | 58 (16.4%) | 48 (13.6%) | 46 (13.0%) | 51 (14.4%) | 42 (11.9%) | 109 (30.8%) | 354 |
| Club instructor / coach | 69 (13.9%) | 78 (15.7%) | 59 (11.9%) | 69 (13.9%) | 62 (12.5%) | 159 (32.1%) | 496 |
| Platform administrator | 34 (18.5%) | 21 (11.4%) | 23 (12.5%) | 25 (13.6%) | 26 (14.1%) | 55 (29.9%) | 184 |
| Other | 9 (12.5%) | 10 (13.9%) | 7 (9.7%) | 11 (15.3%) | 11 (15.3%) | 24 (33.3%) | 72 |

**Panel B. Profile membership by years of platform use**

| **Category** | **Profile 1** | **Profile 2** | **Profile 3** | **Profile 4** | **Profile 5** | **Profile 6** | **Total** |
| --- | --- | --- | --- | --- | --- | --- | --- |
| Less than 1 year | 60 (14.4%) | 60 (14.4%) | 58 (13.9%) | 66 (15.8%) | 49 (11.7%) | 125 (29.9%) | 418 |
| 1–2 years | 90 (12.9%) | 100 (14.3%) | 80 (11.4%) | 104 (14.9%) | 87 (12.4%) | 238 (34.0%) | 699 |
| 3–4 years | 84 (15.9%) | 76 (14.4%) | 75 (14.2%) | 62 (11.8%) | 69 (13.1%) | 161 (30.6%) | 527 |
| 5 years or more | 31 (12.8%) | 45 (18.5%) | 31 (12.8%) | 30 (12.3%) | 30 (12.3%) | 76 (31.3%) | 243 |

**Panel C. Profile membership by frequency of platform use**

| **Category** | **Profile 1** | **Profile 2** | **Profile 3** | **Profile 4** | **Profile 5** | **Profile 6** | **Total** |
| --- | --- | --- | --- | --- | --- | --- | --- |
| Rarely | 17 (13.4%) | 20 (15.7%) | 15 (11.8%) | 20 (15.7%) | 15 (11.8%) | 40 (31.5%) | 127 |
| Several times per month | 44 (10.5%) | 50 (11.9%) | 61 (14.5%) | 59 (14.0%) | 65 (15.4%) | 142 (33.7%) | 421 |
| Several times per week | 125 (15.2%) | 116 (14.1%) | 105 (12.8%) | 118 (14.3%) | 100 (12.2%) | 259 (31.5%) | 823 |
| Almost every day | 79 (15.3%) | 95 (18.4%) | 63 (12.2%) | 65 (12.6%) | 55 (10.7%) | 159 (30.8%) | 516 |

**Panel D. Profile membership by main platform function used**

| **Category** | **Profile 1** | **Profile 2** | **Profile 3** | **Profile 4** | **Profile 5** | **Profile 6** | **Total** |
| --- | --- | --- | --- | --- | --- | --- | --- |
| Course selection management | 47 (12.2%) | 47 (12.2%) | 47 (12.2%) | 61 (15.8%) | 60 (15.5%) | 124 (32.1%) | 386 |
| Attendance management | 75 (16.2%) | 78 (16.9%) | 60 (13.0%) | 57 (12.3%) | 65 (14.1%) | 127 (27.5%) | 462 |
| Performance assessment | 45 (14.5%) | 52 (16.8%) | 42 (13.5%) | 39 (12.6%) | 31 (10.0%) | 101 (32.6%) | 310 |
| Venue / club coordination | 43 (12.5%) | 46 (13.4%) | 49 (14.2%) | 49 (14.2%) | 40 (11.6%) | 117 (34.0%) | 344 |
| Comprehensive teaching management | 55 (14.3%) | 58 (15.1%) | 46 (11.9%) | 56 (14.5%) | 39 (10.1%) | 131 (34.0%) | 385 |

Note. Percentages are calculated within each subgroup category. Pearson chi-square tests were exploratory; Cramér’s V is reported as the effect-size index. All expected cell counts were greater than five. Profile 1 = low-SQ/low-PEOU; Profile 2 = moderate-SQ/high-PEOU; Profile 3 = low-SQ/high-PEOU; Profile 4 = moderate-SQ/low-PEOU; Profile 5 = high-SQ/low-PEOU; Profile 6 = high-SQ/high-PEOU. SQ = digital teaching management system quality; PEOU = perceived ease of use.
