# Supplementary material for: Linking digital teaching management platform quality to perceived net benefits: a hybrid SEM–LPA study in university sports club-based teaching
Source: Front Psychol. 2026 Aug 5;17:1903344. doi: 10.3389/fpsyg.2026.1903344 (PMC13485549; doi:10.3389/fpsyg.2026.1903344)
Supplement: Supplementary file 2 [file Table_3.docx]

**Data Dictionary**

*Linking Digital Teaching Management Platform Quality to Perceived Net Benefits: A Hybrid SEM-LPA Study in University Sports Club-Based Teaching*

## Dataset Overview

| **Field** | **Description** |
| --- | --- |
| Dataset | Cleaned analytic dataset |
| Unit of analysis | Individual adult platform user |
| Number of records | 1,887 |
| Number of variables | 34 |
| Data structure | One row represents one valid survey respondent. |
| Missing data | No missing values are present in the submitted analytic dataset. |
| Response scale | Item-level variables use a five-point Likert scale: 1 = Strongly disagree; 2 = Disagree; 3 = Neutral; 4 = Agree; 5 = Strongly agree. |
| Scoring direction | All item variables are positively worded and positively scored; higher scores indicate higher levels of the corresponding construct. |
| Identifier | ID is an anonymized sequential record number and is not a personal identifier. |

## Construct Abbreviations

| **Abbreviation** | **Construct** | **Definition** | **Item Indicators** | **Composite Variable** |
| --- | --- | --- | --- | --- |
| SQ | Digital teaching management system quality | Technical and functional quality of the digital teaching management platform as perceived by users. | 7 item indicators | X_SQ |
| PEOU | Perceived ease of use | The degree to which users perceive the platform as requiring little effort to use. | 4 item indicators | M1_PEOU |
| PU | Perceived usefulness | The degree to which users perceive the platform as improving teaching management performance. | 4 item indicators | M2_PU |
| SAT | User satisfaction | Users' overall affective and evaluative response to their experience with the platform. | 3 item indicators | M3_SAT |
| NB | Teaching management net benefits | Perceived value of the platform for sports club-based teaching management. | 10 item indicators | Y_NB |

## Construct Scoring

| **Composite Variable** | **Construct** | **Item Variables** | **Number of Items** | **Scoring Rule** | **Interpretation** |
| --- | --- | --- | --- | --- | --- |
| X_SQ | Digital teaching management system quality | X1_SQ_1 to X1_SQ_7 | 7 | Arithmetic mean of all seven item responses. | Higher values indicate higher perceived system quality. |
| M1_PEOU | Perceived ease of use | M1_PEOU_1 to M1_PEOU_4 | 4 | Arithmetic mean of all four item responses. | Higher values indicate greater perceived ease of use. |
| M2_PU | Perceived usefulness | M2_PU_1 to M2_PU_4 | 4 | Arithmetic mean of all four item responses. | Higher values indicate greater perceived usefulness. |
| M3_SAT | User satisfaction | M3_SAT_1 to M3_SAT_3 | 3 | Arithmetic mean of all three item responses. | Higher values indicate greater user satisfaction. |
| Y_NB | Teaching management net benefits | Y_NB_1 to Y_NB_10 | 10 | Arithmetic mean of all ten item responses. | Higher values indicate greater perceived teaching management net benefits. |

## Demographic and Platform Use Coding

| **Variable** | **Code variable** | **Coding / categories** |
| --- | --- | --- |
| Gender | Gender_code | 1 = Male; 2 = Female; 3 = Other / Prefer not to say. |
| Age | Age_code | 1 = <= 30 years; 2 = 31-40 years; 3 = 41-50 years; 4 = >= 51 years. |
| Education_level | Education_level_code | 1 = Bachelor's degree or below; 2 = Master's degree; 3 = Doctoral degree. |
| Professional_title | Professional_title_code | 1 = Teaching assistant; 2 = Lecturer; 3 = Associate professor; 4 = Professor; 5 = Administrative staff / Other professional title. |
| Platform_role | Platform_role_code | 1 = Physical education teacher; 2 = Teaching administrator; 3 = Club instructor / coach; 4 = Platform administrator; 5 = Other. |
| Teaching_experience | Teaching_experience_code | 1 = Less than 5 years; 2 = 5-10 years; 3 = 11-20 years; 4 = More than 20 years. |
| Platform_use_years | Platform_use_years_code | 1 = Less than 1 year; 2 = 1-2 years; 3 = 3-4 years; 4 = 5 years or more. |
| Platform_use_frequency | Platform_use_frequency_code | 1 = Rarely; 2 = Several times per month; 3 = Several times per week; 4 = Almost every day. |
| Main_platform_function | Main_platform_function_code | 1 = Course selection management; 2 = Attendance management; 3 = Performance assessment; 4 = Venue / club coordination; 5 = Comprehensive teaching management. |

## Variable-Level Data Dictionary

| **Variable Name** | **Construct / Domain** | **Variable Label** | **Analytic Role** | **Type** | **Coding / Valid Values** | **Derivation / Scoring** | **Observed Range** | **Missing Values** |
| --- | --- | --- | --- | --- | --- | --- | --- | --- |
| Y_NB | Teaching management net benefits | Composite score for perceived teaching management net benefits. | Outcome variable; LPA distal outcome | Continuous numeric | Mean score on a 1-5 scale; higher scores indicate greater perceived net benefits. | Arithmetic mean of Y_NB_1 to Y_NB_10. | 1.40-5.00 | 0 |
| M3_SAT | User satisfaction | Composite score for user satisfaction. | Sequential mediator 3; LPA distal outcome | Continuous numeric | Mean score on a 1-5 scale; higher scores indicate greater user satisfaction. | Arithmetic mean of M3_SAT_1 to M3_SAT_3. | 1.00-5.00 | 0 |
| M2_PU | Perceived usefulness | Composite score for perceived usefulness. | Sequential mediator 2; LPA distal outcome | Continuous numeric | Mean score on a 1-5 scale; higher scores indicate greater perceived usefulness. | Arithmetic mean of M2_PU_1 to M2_PU_4. | 1.00-5.00 | 0 |
| M1_PEOU | Perceived ease of use | Composite score for perceived ease of use. | Sequential mediator 1; LPA profile indicator | Continuous numeric | Mean score on a 1-5 scale; higher scores indicate greater perceived ease of use. | Arithmetic mean of M1_PEOU_1 to M1_PEOU_4. | 1.00-5.00 | 0 |
| X_SQ | Digital teaching management system quality | Composite score for perceived system quality. | Primary predictor; LPA profile indicator | Continuous numeric | Mean score on a 1-5 scale; higher scores indicate higher perceived system quality. | Arithmetic mean of X1_SQ_1 to X1_SQ_7. | 1.43-5.00 | 0 |
| Y_NB_1 | Teaching management net benefits | Item 1 measuring perceived net benefits of the platform for sports club-based teaching management. | Observed item indicator | Ordinal numeric | 1 = Strongly disagree; 2 = Disagree; 3 = Neutral; 4 = Agree; 5 = Strongly agree. | Raw item response; positively scored; no reverse coding. | 1.00-5.00 | 0 |
| Y_NB_2 | Teaching management net benefits | Item 2 measuring perceived net benefits of the platform for sports club-based teaching management. | Observed item indicator | Ordinal numeric | 1 = Strongly disagree; 2 = Disagree; 3 = Neutral; 4 = Agree; 5 = Strongly agree. | Raw item response; positively scored; no reverse coding. | 1.00-5.00 | 0 |
| Y_NB_3 | Teaching management net benefits | Item 3 measuring perceived net benefits of the platform for sports club-based teaching management. | Observed item indicator | Ordinal numeric | 1 = Strongly disagree; 2 = Disagree; 3 = Neutral; 4 = Agree; 5 = Strongly agree. | Raw item response; positively scored; no reverse coding. | 1.00-5.00 | 0 |
| Y_NB_4 | Teaching management net benefits | Item 4 measuring perceived net benefits of the platform for sports club-based teaching management. | Observed item indicator | Ordinal numeric | 1 = Strongly disagree; 2 = Disagree; 3 = Neutral; 4 = Agree; 5 = Strongly agree. | Raw item response; positively scored; no reverse coding. | 1.00-5.00 | 0 |
| Y_NB_5 | Teaching management net benefits | Item 5 measuring perceived net benefits of the platform for sports club-based teaching management. | Observed item indicator | Ordinal numeric | 1 = Strongly disagree; 2 = Disagree; 3 = Neutral; 4 = Agree; 5 = Strongly agree. | Raw item response; positively scored; no reverse coding. | 1.00-5.00 | 0 |
| Y_NB_6 | Teaching management net benefits | Item 6 measuring perceived net benefits of the platform for sports club-based teaching management. | Observed item indicator | Ordinal numeric | 1 = Strongly disagree; 2 = Disagree; 3 = Neutral; 4 = Agree; 5 = Strongly agree. | Raw item response; positively scored; no reverse coding. | 1.00-5.00 | 0 |
| Y_NB_7 | Teaching management net benefits | Item 7 measuring perceived net benefits of the platform for sports club-based teaching management. | Observed item indicator | Ordinal numeric | 1 = Strongly disagree; 2 = Disagree; 3 = Neutral; 4 = Agree; 5 = Strongly agree. | Raw item response; positively scored; no reverse coding. | 1.00-5.00 | 0 |
| Y_NB_8 | Teaching management net benefits | Item 8 measuring perceived net benefits of the platform for sports club-based teaching management. | Observed item indicator | Ordinal numeric | 1 = Strongly disagree; 2 = Disagree; 3 = Neutral; 4 = Agree; 5 = Strongly agree. | Raw item response; positively scored; no reverse coding. | 1.00-5.00 | 0 |
| Y_NB_9 | Teaching management net benefits | Item 9 measuring perceived net benefits of the platform for sports club-based teaching management. | Observed item indicator | Ordinal numeric | 1 = Strongly disagree; 2 = Disagree; 3 = Neutral; 4 = Agree; 5 = Strongly agree. | Raw item response; positively scored; no reverse coding. | 1.00-5.00 | 0 |
| Y_NB_10 | Teaching management net benefits | Item 10 measuring perceived net benefits of the platform for sports club-based teaching management. | Observed item indicator | Ordinal numeric | 1 = Strongly disagree; 2 = Disagree; 3 = Neutral; 4 = Agree; 5 = Strongly agree. | Raw item response; positively scored; no reverse coding. | 1.00-5.00 | 0 |
| M3_SAT_1 | User satisfaction | Item 1 measuring overall satisfaction with the platform. | Observed item indicator | Ordinal numeric | 1 = Strongly disagree; 2 = Disagree; 3 = Neutral; 4 = Agree; 5 = Strongly agree. | Raw item response; positively scored; no reverse coding. | 1.00-5.00 | 0 |
| M3_SAT_2 | User satisfaction | Item 2 measuring overall satisfaction with the platform. | Observed item indicator | Ordinal numeric | 1 = Strongly disagree; 2 = Disagree; 3 = Neutral; 4 = Agree; 5 = Strongly agree. | Raw item response; positively scored; no reverse coding. | 1.00-5.00 | 0 |
| M3_SAT_3 | User satisfaction | Item 3 measuring overall satisfaction with the platform. | Observed item indicator | Ordinal numeric | 1 = Strongly disagree; 2 = Disagree; 3 = Neutral; 4 = Agree; 5 = Strongly agree. | Raw item response; positively scored; no reverse coding. | 1.00-5.00 | 0 |
| M2_PU_1 | Perceived usefulness | Item 1 measuring perceived usefulness of the platform for teaching management tasks. | Observed item indicator | Ordinal numeric | 1 = Strongly disagree; 2 = Disagree; 3 = Neutral; 4 = Agree; 5 = Strongly agree. | Raw item response; positively scored; no reverse coding. | 1.00-5.00 | 0 |
| M2_PU_2 | Perceived usefulness | Item 2 measuring perceived usefulness of the platform for teaching management tasks. | Observed item indicator | Ordinal numeric | 1 = Strongly disagree; 2 = Disagree; 3 = Neutral; 4 = Agree; 5 = Strongly agree. | Raw item response; positively scored; no reverse coding. | 1.00-5.00 | 0 |
| M2_PU_3 | Perceived usefulness | Item 3 measuring perceived usefulness of the platform for teaching management tasks. | Observed item indicator | Ordinal numeric | 1 = Strongly disagree; 2 = Disagree; 3 = Neutral; 4 = Agree; 5 = Strongly agree. | Raw item response; positively scored; no reverse coding. | 1.00-5.00 | 0 |
| M2_PU_4 | Perceived usefulness | Item 4 measuring perceived usefulness of the platform for teaching management tasks. | Observed item indicator | Ordinal numeric | 1 = Strongly disagree; 2 = Disagree; 3 = Neutral; 4 = Agree; 5 = Strongly agree. | Raw item response; positively scored; no reverse coding. | 1.00-5.00 | 0 |
| M1_PEOU_1 | Perceived ease of use | Item 1 measuring perceived effortlessness of using the platform. | Observed item indicator | Ordinal numeric | 1 = Strongly disagree; 2 = Disagree; 3 = Neutral; 4 = Agree; 5 = Strongly agree. | Raw item response; positively scored; no reverse coding. | 1.00-5.00 | 0 |
| M1_PEOU_2 | Perceived ease of use | Item 2 measuring perceived effortlessness of using the platform. | Observed item indicator | Ordinal numeric | 1 = Strongly disagree; 2 = Disagree; 3 = Neutral; 4 = Agree; 5 = Strongly agree. | Raw item response; positively scored; no reverse coding. | 1.00-5.00 | 0 |
| M1_PEOU_3 | Perceived ease of use | Item 3 measuring perceived effortlessness of using the platform. | Observed item indicator | Ordinal numeric | 1 = Strongly disagree; 2 = Disagree; 3 = Neutral; 4 = Agree; 5 = Strongly agree. | Raw item response; positively scored; no reverse coding. | 1.00-5.00 | 0 |
| M1_PEOU_4 | Perceived ease of use | Item 4 measuring perceived effortlessness of using the platform. | Observed item indicator | Ordinal numeric | 1 = Strongly disagree; 2 = Disagree; 3 = Neutral; 4 = Agree; 5 = Strongly agree. | Raw item response; positively scored; no reverse coding. | 1.00-5.00 | 0 |
| X1_SQ_1 | Digital teaching management system quality | Item 1 measuring perceived technical and functional quality of the platform. | Observed item indicator | Ordinal numeric | 1 = Strongly disagree; 2 = Disagree; 3 = Neutral; 4 = Agree; 5 = Strongly agree. | Raw item response; positively scored; no reverse coding. | 1.00-5.00 | 0 |
| X1_SQ_2 | Digital teaching management system quality | Item 2 measuring perceived technical and functional quality of the platform. | Observed item indicator | Ordinal numeric | 1 = Strongly disagree; 2 = Disagree; 3 = Neutral; 4 = Agree; 5 = Strongly agree. | Raw item response; positively scored; no reverse coding. | 1.00-5.00 | 0 |
| X1_SQ_3 | Digital teaching management system quality | Item 3 measuring perceived technical and functional quality of the platform. | Observed item indicator | Ordinal numeric | 1 = Strongly disagree; 2 = Disagree; 3 = Neutral; 4 = Agree; 5 = Strongly agree. | Raw item response; positively scored; no reverse coding. | 1.00-5.00 | 0 |
| X1_SQ_4 | Digital teaching management system quality | Item 4 measuring perceived technical and functional quality of the platform. | Observed item indicator | Ordinal numeric | 1 = Strongly disagree; 2 = Disagree; 3 = Neutral; 4 = Agree; 5 = Strongly agree. | Raw item response; positively scored; no reverse coding. | 1.00-5.00 | 0 |
| X1_SQ_5 | Digital teaching management system quality | Item 5 measuring perceived technical and functional quality of the platform. | Observed item indicator | Ordinal numeric | 1 = Strongly disagree; 2 = Disagree; 3 = Neutral; 4 = Agree; 5 = Strongly agree. | Raw item response; positively scored; no reverse coding. | 1.00-5.00 | 0 |
| X1_SQ_6 | Digital teaching management system quality | Item 6 measuring perceived technical and functional quality of the platform. | Observed item indicator | Ordinal numeric | 1 = Strongly disagree; 2 = Disagree; 3 = Neutral; 4 = Agree; 5 = Strongly agree. | Raw item response; positively scored; no reverse coding. | 1.00-5.00 | 0 |
| X1_SQ_7 | Digital teaching management system quality | Item 7 measuring perceived technical and functional quality of the platform. | Observed item indicator | Ordinal numeric | 1 = Strongly disagree; 2 = Disagree; 3 = Neutral; 4 = Agree; 5 = Strongly agree. | Raw item response; positively scored; no reverse coding. | 1.00-5.00 | 0 |
| ID | Record information | Anonymized sequential record identifier. | Identifier | Integer | 1 to 1,887 | Sequential record number assigned to each valid case. | 1.00-1887.00 | 0 |

## Variable Descriptive Statistics

| **Variable Name** | **N** | **Missing Values** | **Observed Minimum** | **Observed Maximum** | **Mean** | **Standard Deviation** | **Number of Unique Values** |
| --- | --- | --- | --- | --- | --- | --- | --- |
| Y_NB | 1887 | 0 | 1.400000 | 5.000000 | 3.591468 | 0.907297 | 37 |
| M3_SAT | 1887 | 0 | 1.000000 | 5.000000 | 3.358417 | 1.010004 | 13 |
| M2_PU | 1887 | 0 | 1.000000 | 5.000000 | 3.231055 | 1.009600 | 17 |
| M1_PEOU | 1887 | 0 | 1.000000 | 5.000000 | 3.388050 | 1.041723 | 17 |
| X_SQ | 1887 | 0 | 1.428571 | 5.000000 | 3.582482 | 0.943170 | 26 |
| Y_NB_1 | 1887 | 0 | 1.000000 | 5.000000 | 3.542130 | 1.062321 | 5 |
| Y_NB_2 | 1887 | 0 | 1.000000 | 5.000000 | 3.585586 | 1.043570 | 5 |
| Y_NB_3 | 1887 | 0 | 1.000000 | 5.000000 | 3.602544 | 1.089794 | 5 |
| Y_NB_4 | 1887 | 0 | 1.000000 | 5.000000 | 3.716481 | 1.178933 | 5 |
| Y_NB_5 | 1887 | 0 | 1.000000 | 5.000000 | 3.461049 | 1.266856 | 5 |
| Y_NB_6 | 1887 | 0 | 1.000000 | 5.000000 | 3.803392 | 1.237034 | 5 |
| Y_NB_7 | 1887 | 0 | 1.000000 | 5.000000 | 3.708532 | 1.094596 | 5 |
| Y_NB_8 | 1887 | 0 | 1.000000 | 5.000000 | 3.528882 | 1.191205 | 5 |
| Y_NB_9 | 1887 | 0 | 1.000000 | 5.000000 | 3.487546 | 1.207404 | 5 |
| Y_NB_10 | 1887 | 0 | 1.000000 | 5.000000 | 3.478537 | 1.179284 | 5 |
| M3_SAT_1 | 1887 | 0 | 1.000000 | 5.000000 | 3.199788 | 1.165899 | 5 |
| M3_SAT_2 | 1887 | 0 | 1.000000 | 5.000000 | 3.461579 | 1.200690 | 5 |
| M3_SAT_3 | 1887 | 0 | 1.000000 | 5.000000 | 3.413884 | 1.213165 | 5 |
| M2_PU_1 | 1887 | 0 | 1.000000 | 5.000000 | 3.088500 | 1.191215 | 5 |
| M2_PU_2 | 1887 | 0 | 1.000000 | 5.000000 | 3.301007 | 1.209065 | 5 |
| M2_PU_3 | 1887 | 0 | 1.000000 | 5.000000 | 3.296237 | 1.222232 | 5 |
| M2_PU_4 | 1887 | 0 | 1.000000 | 5.000000 | 3.238474 | 1.221643 | 5 |
| M1_PEOU_1 | 1887 | 0 | 1.000000 | 5.000000 | 3.453100 | 1.201713 | 5 |
| M1_PEOU_2 | 1887 | 0 | 1.000000 | 5.000000 | 3.397986 | 1.204030 | 5 |
| M1_PEOU_3 | 1887 | 0 | 1.000000 | 5.000000 | 3.332273 | 1.193537 | 5 |
| M1_PEOU_4 | 1887 | 0 | 1.000000 | 5.000000 | 3.368839 | 1.245839 | 5 |
| X1_SQ_1 | 1887 | 0 | 1.000000 | 5.000000 | 3.480657 | 1.198055 | 5 |
| X1_SQ_2 | 1887 | 0 | 1.000000 | 5.000000 | 3.519873 | 1.215620 | 5 |
| X1_SQ_3 | 1887 | 0 | 1.000000 | 5.000000 | 3.624801 | 1.084050 | 5 |
| X1_SQ_4 | 1887 | 0 | 1.000000 | 5.000000 | 3.541600 | 1.272201 | 5 |
| X1_SQ_5 | 1887 | 0 | 1.000000 | 5.000000 | 3.661897 | 1.071735 | 5 |
| X1_SQ_6 | 1887 | 0 | 1.000000 | 5.000000 | 3.699523 | 1.104946 | 5 |
| X1_SQ_7 | 1887 | 0 | 1.000000 | 5.000000 | 3.549020 | 1.225224 | 5 |
| ID | 1887 | 0 | 1.000000 | 1887.000000 | 944.000000 | 544.874297 | 1887 |
